# Optimal site for applying transcutaneous bilirubinometer as an outpatient screening tool for neonatal jaundice: a comparison between the sternum and forehead

**DOI:** 10.3389/fped.2024.1446524

**Published:** 2024-12-11

**Authors:** Emily Zhang, Tzong-Jin Wu, Mark L. Hudak, Ke Yan, Ru-Jeng Teng

**Affiliations:** ^1^ College of Human Ecology, Cornell University, Ithaca, NY, United States; ^2^ Department of Pediatrics, Division of Neonatology, Medical College of Wisconsin, Wauwatosa, WI, United States; ^3^ Department of Pediatrics, Division of Neonatology, University of Florida Health Science Center at Jacksonville, Jacksonville, FL, United States; ^4^ Department of Pediatrics, Division of Quantitative Health Sciences, Medical College of Wisconsin, Wauwatosa, WI, United States

**Keywords:** bilirubin, neonatal jaundice, transcutaneous bilirubinometer, sternum, forehead, outpatient follow-up

## Abstract

**Background:**

The gold standard for assessing neonatal jaundice (NJ) is the serum total serum bilirubin (TSB) level by the diazo method. A transcutaneous bilirubinometer (TCB) provides a convenient, noninvasive readout within minutes. The reliability of TCB as the diagnostic tool and the proper site for TCB measurement remains unsettled.

**Objectives:**

This study aimed to (1) evaluate the reliability of TCB in the NJ outpatient management and (2) identify a better site to obtain TCB readings.

**Methods:**

This retrospective study examines data collected prospectively over 15 months at a level III facility. Parents were advised to bring their neonates back to our nursery if neonates were judged to be at risk for NJ or poor weight gain, and a follow-up with the primary practitioner was not available. Those who had received phototherapy or sustained forehead bruising were excluded from the analysis. Blood was collected immediately after TCB readings for TSB measurement using the di-azo method. The primary endpoint was admission for treatment according to the AAP 2004 guidelines. A mixed-effects model was used to assess the correlation of forehead TCB (TCB-_f_) or sternal TCB (TCB-_s_) with TSB by adjusting for age at measurement (hours), gestational age (GA), sex, and race. Repeated Measure Receiver Operator Characteristic (ROC) curves were constructed for TCB readings against the hospital admission, and the cutoffs for each method were selected to balance the sensitivity and specificity.

**Results:**

There were 500 visits for 350 neonates, including 136 females, 114 white, 134 black, 71 Hispanic, and 30 Asian. The mean GA was 38.5 weeks [standard deviation (SD) = 1.6], and the mean body weight (BW) was 3,238 g (SD = 506). Forty-five (12.9%) neonates were admitted for phototherapy or blood exchange transfusion according to the TSB levels. Only 43 admitted neonates had all three measurements. Assuming TCB has the same reading as TSB, 30 out of 43 (69.8%) and 20 out of 43 (46.5%) neonates would be sent home if only TCB-_f_ and TCB-_s_ were used, respectively. TCB_f_ has a trend of underestimating the necessity of hospitalization compared to TCB_s_ (*p* = 0.092 by McNemar test). After adjusting for age of measurement, GA, sex, and race, both TCB-_f_ and TCB-_s_ readings positively correlated with TSB (*p* < 0.0001). Using repeated measure ROC, with hospital admission for treatment as the primary outcome, the area under the curve (AUC) for TCB-_f_ was 0.79 (95% CI: 0.71–0.86), and AUC for TCB-_s_ was 0.86 (95% CI: 0.81–0.92). A cutoff of 14.3 for TCB-_s_ gave a sensitivity of 81% and a specificity of 78%. A cutoff of 12.6 for TCB-_f_ gave a sensitivity of 80% and a specificity of 65%.

**Conclusions:**

TCB measurements can discriminate well in predicting admission for NJ treatment in our nursery but tend to underestimate the severity. The sternum is a better site for TCB measurements. We must point out that more than 40% of neonates who should be admitted for NJ management would be sent home if TSB were not obtained simultaneously. We recommend adjusting TCB readings according to unit-based calibration before clinical implementation.

## Introduction

Neonatal jaundice (NJ) is a prevalent clinical condition in infants worldwide; approximately 60%–80% of newborns develop clinical jaundice in the first week after birth (TSB or total serum bilirubin above 2 mg/dl) (1, 2). The most severe risk of untreated jaundice is kernicterus, characterized by long-term neurologic damage from bilirubin deposits in the basal ganglion, resulting in bilirubin-induced neurologic dysfunction (BIND), cerebral palsy or even death (3, 4).

Because of these risks, The American Academy of Pediatrics (AAP) guidelines in 2004 recommended screening for NJ and its risk factors before discharge from the hospital (5), with an update in 2022 (6). The study was conducted before the 2022 version, and the clinical decisions were based on the 2004 guidelines. However, most full-term infants are discharged at 2–3 days of age (7), while bilirubin levels still rise for most of them (8). Thus, AAP recommends that health professionals evaluate neonates within two days after discharge from the nursery. This recommendation significantly burdens medical staff, especially on weekends or holidays. Another practical hurdle is that most clinics are not equipped to measure TSB. A convenient but reliable screening tool for NJ with a short turnaround time will benefit practitioners in proper management.

The gold standard for the NJ determination remains the TSB levels using the diazo reaction (9). Blood sampling to determine TSB is done through a phlebotomy or heel-prick procedure, which is invasive and painful. Moreover, the turnaround time for the TSB result may take hours or longer, depending on the laboratory services’ location and the medical staff's availability, which can inconvenience parents and busy medical professionals.

Over the past two decades, studies have suggested that the methodology of transcutaneous bilirubin (TCB) reading could reliably replace the need to obtain TSB in a well-controlled environment where designated personnel could obtain TCB readings, thus reducing unnecessary blood work (9). Theoretically, TCB readings will not be affected by the skin pigmentation. For transcutaneous devices, multiple factors, including assessment sites, can influence the accuracy of bilirubin measurement. Studies comparing chest or forehead readings have found differences in their correlation with TSB (10–12). However, the current literature has mixed findings depending on patient characteristics, clinical practice style, and hospital settings (13–15).

This study aimed to compare TCB-_s_ (sternum) and TCB-_f_ (forehead) in an outpatient setting, where multiple practitioners are involved in the measurements, to see whether TCB can be used more reliably to screen significant NJ in at-risk neonates.

## Material and methods

### Background information

All infants born at the Shands Hospital of the University of Florida at Jacksonville between May 05, 2005, and August 04, 2006, and being cared for in the newborn nursery, with concerns of marginal enteral intake or significant NJ at discharge, were recommended to be seen by the primary practitioner within the next two days. As Jacksonville is the largest city by area in the USA, we offered weight checks and jaundice evaluations in our nursery as an alternative for parents who could not secure an appointment. Neonates treated by phototherapy or had skin bruises on the forehead or sternal area were excluded from our analysis. The hospital policy did not allow premature neonates discharged from its neonatal intensive care unit to be followed in the nursery. The study was conducted as the initial quality assurance procedure for introducing a new diagnostic instrument. The mothers determined the ethnic group assignment.

### Bilirubin measurements and clinical disposition

TCB-_f_ and TCB-_s_ were obtained in a dark room right before the blood work to avoid the potential influence of ambient light (14). BiliCheck® (Respironics, Marietta, GA; presently owned by Philips Healthcare), provided by the Children Miracle Network as a gift to the hospital, was used for this study and calibrated by the manufacturer. Five allied health professionals from the newborn nursery were trained to operate the instrument to cover as many visits as possible. A disposable calibration tip was applied to the detector for each neonate to prevent cross-contamination. An average of five successful readings was provided as the TCB readout. The operators were instructed to obtain TCB-_f_ and TCB-_s_ for every neonate. We did not specify which TCB site should be obtained first. TSB blood samples were collected after obtaining the TCB readings and immediately shielded from light for shipment to the central laboratory within 15 min of collection to determine the values. The TSB levels were determined automatically by the classical diazo method of Jendrassik-Grof (Modular P800, Roche). The machine was routinely calibrated twice daily against the standards provided by the manufacturer. The technician from the central laboratory reported directly to the nursery for TSB results by phone.

### Data collection

All the TCB readings, TSB results, date and time of birth, date and time of visit, sex, self-determined ethnicity by the mothers, and final disposition were recorded on the log as an integral part of our quality control/quality assurance (QA/QI). The physicians determined the hospitalization according to the hour-specific thresholds and risk factors published in the 2004 AAP guidelines. The QI/QA project was performed after receiving approval from the Institutional Review Board (IRB) of the University of Florida Health Science Center at Jacksonville. The data were analyzed retrospectively using the convenient sample. The informed consent was waived per IRB policy.

### Statistical analysis

Descriptive statistics were computed for all the variables, including mean ± SD, ranges for continuous variables, and frequency counts and percentages for categorical variables. Due to each subject having 1–6 visits, the Bland-Altman plot for multiple observations per individual was used to evaluate the agreement between TCB readings and TSB values. We used the folded empirical cumulative distribution (mountain) plot to assess the bias and difference between the two TCB readings against concurrent TSB levels. McNemar's test was used to compare the chance of missing to admit the neonates between TCB-_f_ and TCB-_s_, assuming that TSB is unavailable and TCB reading is the same as TSB level. The mixed-effects model was used to investigate the relationships between TCB-_f_ or TCB-_s_ and TSB and the relationships between TCB-_f_ or TCB-_s_ and race. Repeated measures ROC curves were constructed with admission for jaundice treatment as the dependent variable and TCB-_f_ or TCB-_s_ values as the independent variable. SAS 9.4 (SAS Institute Inc., Cary, NC, USA, 2016) and IBM SPSS Statistics 28 (IBM Corp., Armonk, NY, USA) were used for all the analyses. A *p*-value <0.05 was considered statistically significant. The Bland-Altman plot for multiple observations per individual and the folded empirical cumulative distribution (mountain) plot were generated by the MedCalc® Statistical Software version 22.023 (MedCalc Software Ltd, Ostend, Belgium; https://www.medcalc.org; 2024).

## Results

### Demographics

**Figure 1:**
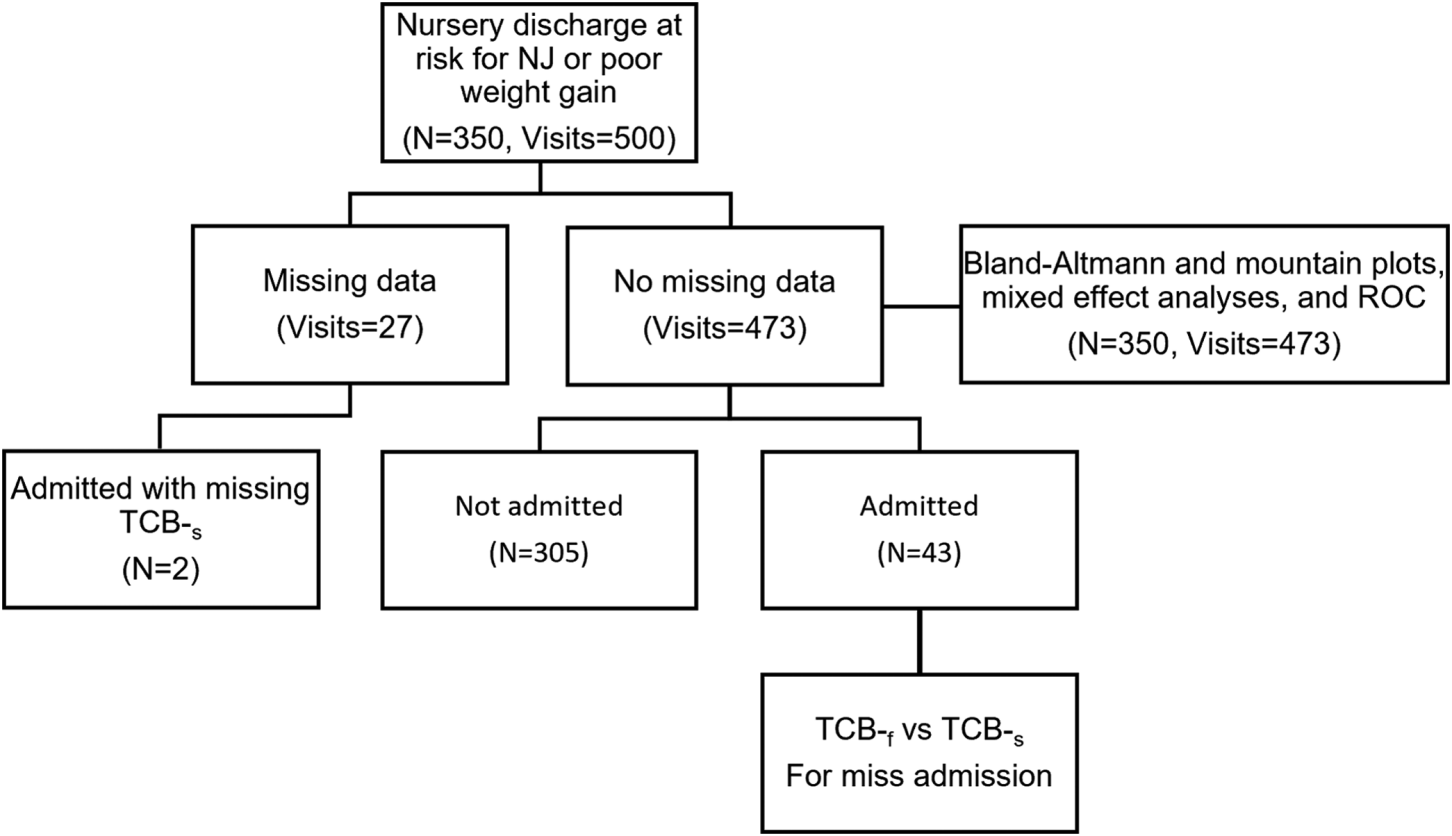
Flowchart of the collected information. There are a total of 350 neonates with 500 visits. Two of the 45 admitted neonates have no transcutaneous readings of the chest (TCB-_s_). Hence, only 43 neonates are included in the McNemar's test. Twenty-seven visits have missing data for transcutaneous readings of the forehead (TCB-_f_) or TCB-_s_, so they are excluded from further analysis.

**Figure 2:**
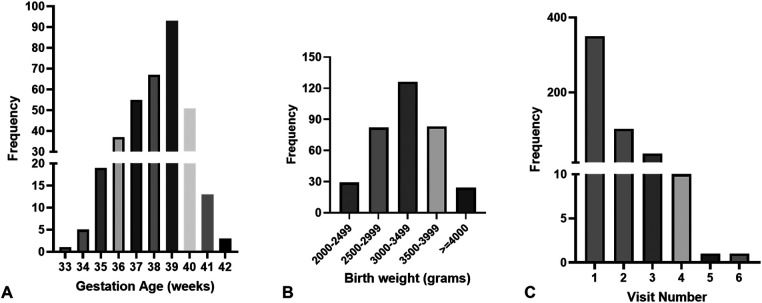
The distribution of participants’ demographic data. **(A)** The gestational ages range between 33 and 42 complete weeks. Only three neonates are born at less than 35 weeks. **(B)** The birth weight distribution. Twenty-eight neonates are born at less than 2,500 g, and twenty-five are born at more than 4,000 g. **(C)** The distribution of visit number per neonate. Most neonates only visit our service once, with eleven visiting more than 3 times.

There were 350 (210 males, 60.7%) neonates, and 500 visits were recorded (Figure 1). The number of visits per infant ranged between 1 and 6. The ethnicity distribution of the infants was 114 Caucasian (32.7%), 134 African American (38.4%), 71 Hispanic (20.3%), and Asian (8.6%). The gestational ages (GA) ranged from 33.0 to 42.3 weeks (mean ± SD: 38.5 ± 1.6 weeks), and birth weights (BW) from 2,029 to 4,550 g (mean ± SD: 3,238.0 ± 506.0 g). Figure 2 shows the distribution of participants’ demographic data. The ranges of TSB, TCB-_f_, and TCB-_s_ were 3.6–33.9 (mean ± SD: 14.6 ± 3.0) mg/dl, 3.7–18.7 (mean ± SD: 12.0 ± 2.6) mg/dl, and 2.7–20.0 (mean ± SD: 12.7 ± 2.6) mg/dl, respectively (Table 1). Forty-five of the 350 (12.9%) neonates were admitted for phototherapy or blood exchange transfusion as judged by the care team according to TSB levels and the 2004 AAP guideline. Six among the 350 neonates (1.7%) were less than 35 weeks gestation, and all needed to be admitted for phototherapy (Figure 2). The forty-five neonates admitted for phototherapy or blood exchange transfusion included 18 Caucasian (40.0%), 16 African American (35.6%), 8 Hispanic (17.8%), and 3 Asian (6.7%). TCB-_f_ and TCB-_s_ for these 45 infants were 8.4–18.7 mg/dl and 10.9–20.0 mg/dl, respectively.

**Table 1:** Result of hospital admission of the 350 neonates and characteristics of the 500 newborn nursery visits.

Variable	*n* (%)
Hospital admission (*n* = 350)	
Yes	45 (12.9%)
No	305 (87.1%)
Visits (*n* = 500)	Mean (SD.)
Infant age (hours)	102.9 (39.8)
TCB_f_	12.0 (2.6)
TCB_s_	12.7 (2.6)
TSB	14.6 (3.0)

TCB_f_, transcutaneous bilirubin level at forehead; TCB_s_, transcutaneous bilirubin level at sternum; TSB, total serum bilirubin.

### The agreement between TCB and TSB

**Figure 3:**
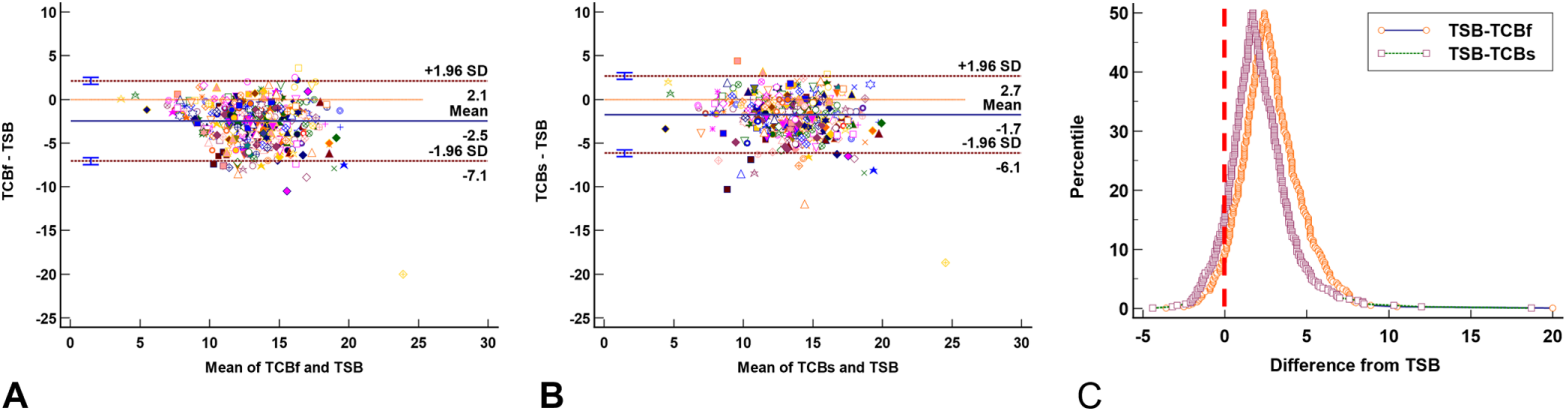
The agreement plots between total serum bilirubin (TSB) levels and transcutaneous bilirubinometer (TCB) readings show TCB readings are generally lower than the corresponding TSB levels. **(A)** Bland-Altman plot for multiple observations per individual shows the TCB-_f_ readings are lower than concurrent TSB levels (−2.5 ± 2.3). **(B)** Bland-Altman plot for multiple observations per individual shows the TCB-_s_ readings are lower than concurrent TSB levels (−1.7 ± 2.2). **(C)** The folded empirical cumulative distribution (mountain) plot shows the distribution of the discrepancies between TCB and TSB. The plot clearly shows a significant difference between the performance of TCB-_f_ and TCB-_s_, with TCB-_s_ being less different from TSB. In **(A)** and **(B)**, each marker's color and shape represents one neonate. The red vertical line in **(C)** represents the identity line between TCB and TSB.

Among the 500 visits, only 473 had complete TSB, TCB-_f_, and TCB-_s_ records. The Bland-Altman plot for multiple observations per individual showed the TCB-_f_ (Figure 3A) and TCB-_s_ (Figure 3B) readings tend to be lower than the concurrent TSB levels by −2.5 ± 2.3 and −1.7 ± 2.2, respectively. The folded empirical cumulative distribution (mountain) plot (Figure 3C) suggested the TCB-_f_ readings were systemically lower than the concurrent TSB levels more than the TCB-_s_. These analyses indicate that TCB-_f_ readings underestimate the severity of the NJ more than TCB-_s_.

### The impact of TCB readings on medical decision

**Figure 4:**
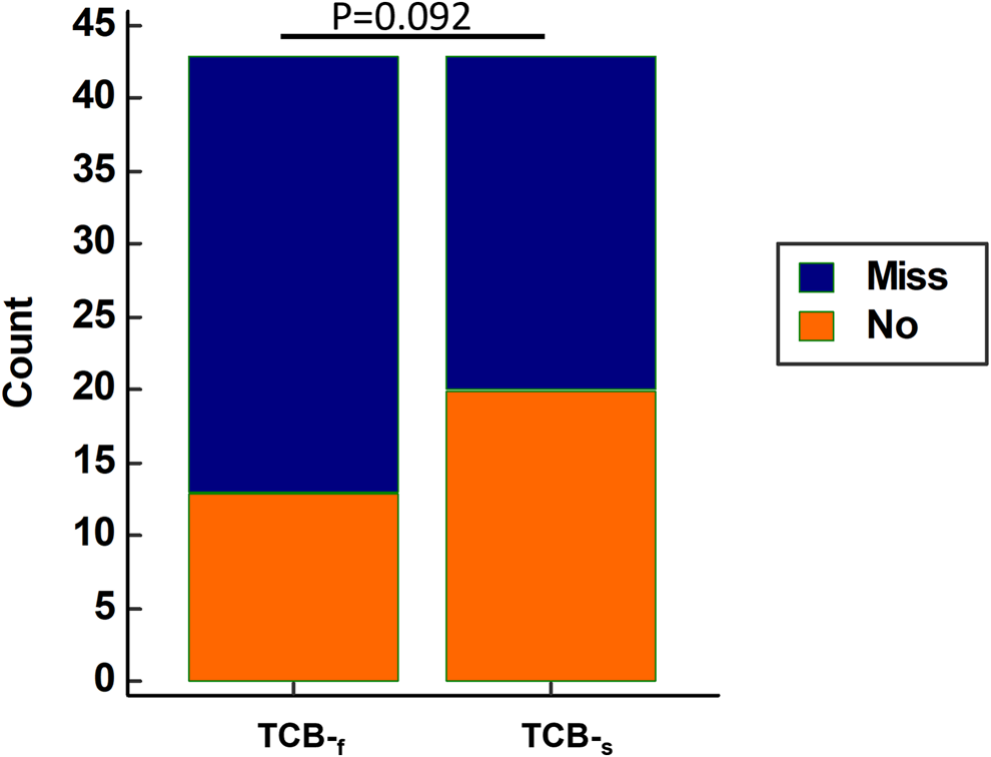
Using raw TCB-_f_ Reading alone tends to misjudge the severity of neonatal jaundice compared to raw TCB-_s_ Reading. For those neonates who should be admitted for phototherapy or blood exchange transfusion, TCB-_f_ alone results in more neonates mistakenly being sent home. Among the 43 admitted neonates with all three measurements, 30 out of 43 (69.8%) and 20 out of 43 (46.5%) neonates would be sent home if only TCB-_f_ and TCB-_s_ were used, respectively. TCB-_f_ has a trend of underestimating the necessity of hospitalization compared to TCB-_s_ (*p* = 0.092 by McNemar test). Blue stack: Neonates who will be mistakenly sent home if concurrent TSB level was not obtained; Red stack: Neonates correctly admitted based on the TCB readings only.

To examine whether clinicians can confidently rely on TCB readings to make correct decisions, we analyzed the data of 43 neonates, out of a total of 45, who were readmitted to the hospital for phototherapy or blood exchange transfusion with all. Data from two neonates were excluded due to the missing TCB-_s_ reading. Surprisingly, 30 out of 43 (69.8%) neonates will be sent home by TCB-_f_ readings if we assume the TCB-_f_ reading is the same as the TSB level. For TCB-_s,_ it was 20 out of 43 (46.5%). Although the performance was better for TCB-_s_ than TCB-f, it did not reach statistical significance (*p* = 0.092 by McNemar test, Figure 4). However, the results were concerning as more than half of the neonates would be sent home if concurrent TSB was not obtained.

### Factors that affect TCB readings

**Figure 5:**
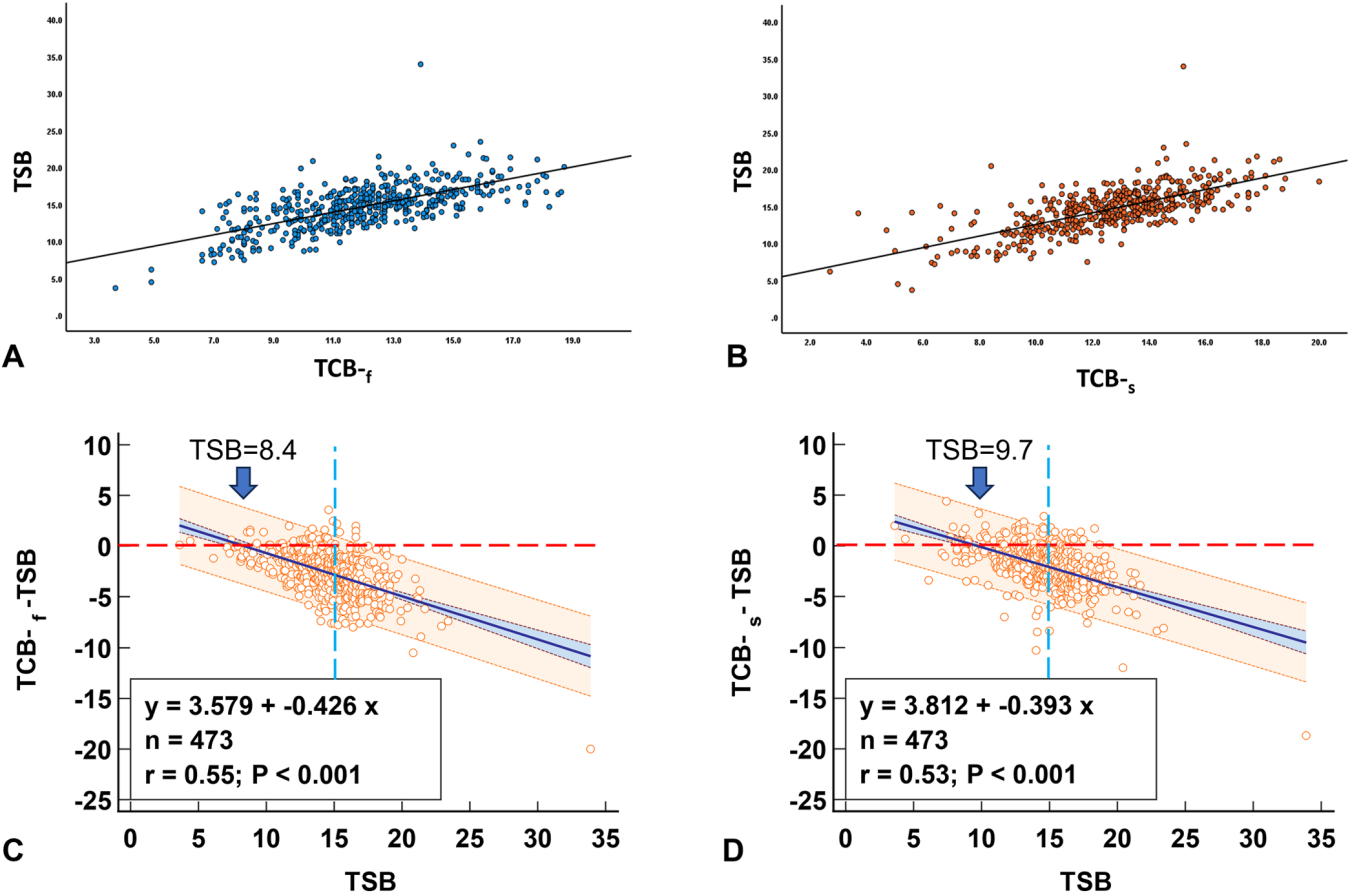
The relationship between TSB and concurrent TCB. When the TSB value is close to the treatment threshold, TCB underestimates the jaundice level. **(A)** Correlation between TSB and TCB-_f_. **(B)** Correlation between TSB and TCB-_s_. **(C)** TCB-_f_ readings start to underestimate the jaundice level at TSB of 8.4. **(D)** TCB-s readings begin to underestimate the jaundice level at TSB of 9.7. Red dash line: identity line; Blue dash line: TSB of 15.0.

**Figure 6:**
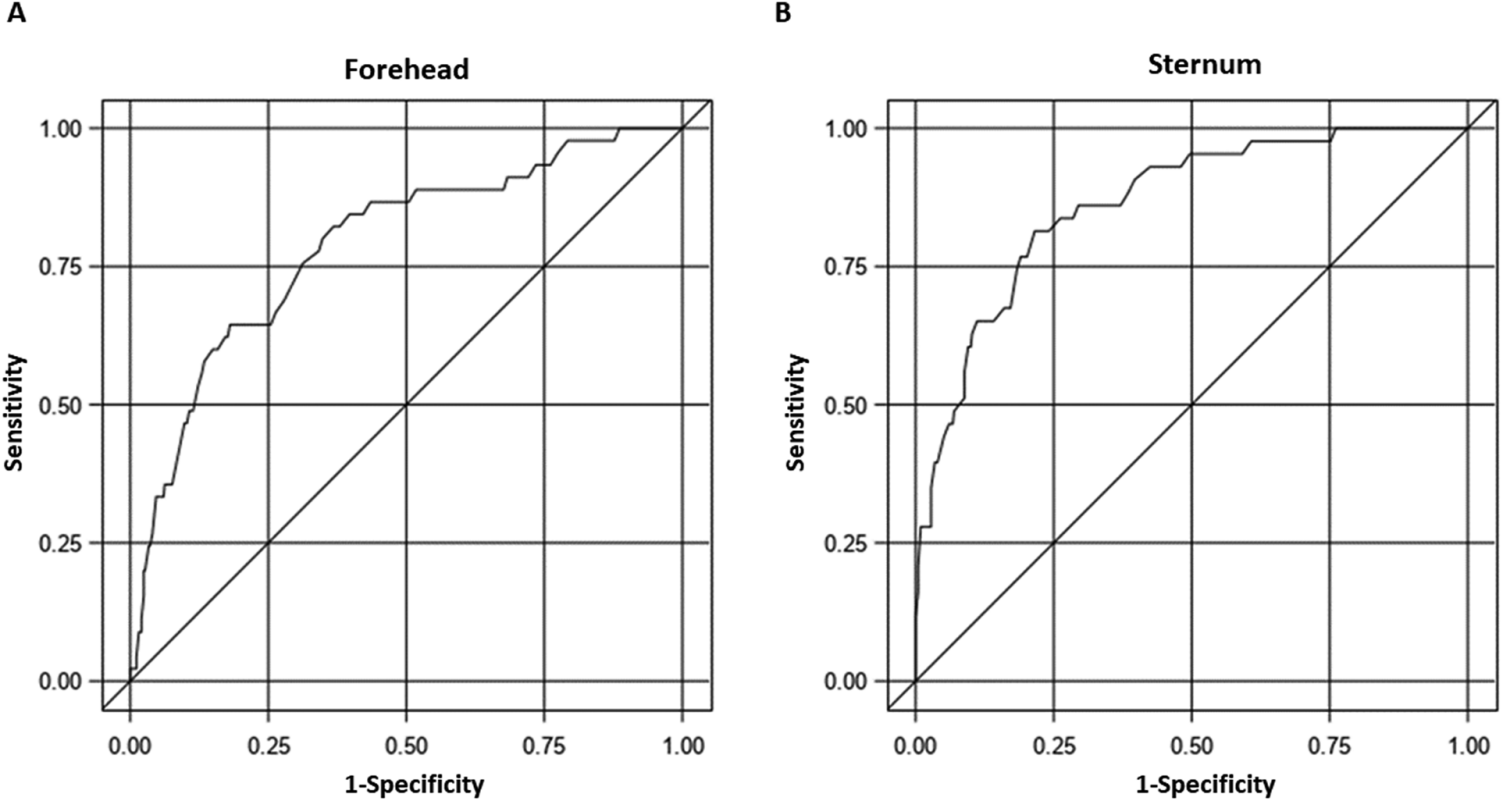
The receiver operating characteristic (ROC) curves for transcutaneous bilirubinometer readings from different sites in predicting admission. **(A)** The AUC for TCB-_f_ is 0.79 (95% confidence interval: 0.71–0.86). **(B)** The AUC for TCB-_s_ is 0.86 (95% confidence interval: 0.81–0.92).

In the multivariable models, after adjusting for various factors, including age at the time of measurement (in hours), GA, sex, and race, both TCBf and TCBs were significantly correlated with TSB (*p* < 0.0001 for both, Figures 5A,B). We plotted TCB-TSB against TSB and revealed that the higher the TSB levels, the more underestimation by the TCB readings (Figures 5C,D). There were significant interactions between GA and TCB-_f_ (*p* = 0.032) but not of GA and TCB-_s_ (*p* = 0.11), which indicated that GA influenced the correlation between TCB-_f_ and TSB but not the correlation between TCB-_s_ and TSB. No associations were observed between TCB-_f_ or TCB-_s_ values and race after adjusting for age at the time of measurement (in hours), GA, and sex (*p* = 0.21 and *p* = 0.25, respectively). Our results indicated that TCB tends to underestimate the severity of NJ when TSB levels are closer to the threshold for clinical management. To predict admission using the repeated measures ROC, the AUC was 0.79 (95% CI: 0.71–0.86) for TCB-_f_ and 0.86 (95% CI: 0.81–0.92) for TCB-_s_. A cutoff of 14.3 for TCB-_s_ gave a sensitivity of 81% and a specificity of 78%. Conversely, for TCB-_f,_ a cutoff of 12.6 showed a sensitivity of 80% and a specificity of 65% (Figure 6).

## Discussion

Although kernicterus occurs at a rate of only 1 to 3 in 100,000 live births in developed countries (16, 17), it can be a significant medicolegal issue and a heavy burden on the family. In some developing countries, severe neonatal jaundice can account for 3% of all admissions (18). The global health impact of severe NJ has led experts to advocate a universal NJ screen policy.

Since the AAP recommendations regarding NJ management were published in 2004 (5), the increase in outpatient follow-ups in delivering hospitals has necessitated a convenient but reliable tool to facilitate the screening process. Before the point-of-care instrument was available, most pediatricians relied on physical examinations to determine which infant should have measured bilirubin levels. AAP recommended that its members use TCB or TSB to make decisions according to the hour-specific, risk-factor-adjusted nomograms (5, 6). Usually, the turnaround time for TSB measurement, either spectrophotometric or diazo method performed in the central laboratory, may take hours, which is inconvenient for practitioners and parents. The introduction of noninvasive TCB technology provides a rational alternative that we can rely on to reduce the workforce needed to monitor NJ.

Reliable bilirubin measurement is essential for appropriate NJ management. With the potential of jaundice to cause severe consequences in neonates, mismanaging a case is highly undesirable. Using a device with a high sensitivity in detecting jaundice is also crucial for reducing the liability of medical decision-makers. Simultaneously, the device must have a desirable level of specificity because overdiagnosis can lead to unnecessary admission and burden to parents. Our data (Figures 3, 5) corroborate other reports regarding the excellent correlation between TCB and TSB, regardless of the skin sites (19). However, our data also showed that at high TSB levels, TCB will underestimate the severity of NJ. This underestimation is especially concerning when the jaundice levels reach the threshold for phototherapy or exchange transfusion. Our study provided a chance to compare TCB-_f_ and TCB-_s_ side-by-side. Although TCB-_s_ outperformed the TCB-f (Figures 3, 4, 6) in our research, it is concerning that more than 50% of NJ neonates who should be admitted for management would be sent home if TSB was not obtained (Figure 4).

We cautioned against using TCB alone when deciding on NJ management. Using a repeated measure ROC curve, we examined the sensitivity and specificity of TCB readings with the Bilicheck®. The ROC curve indicates that setting the cutoff at 14.3 for TCB-_s_ achieves high sensitivity (81%) and specificity (78%). However, maintaining a similar sensitivity (80%) for the TCB-_f_ and selecting the cutoff of 12.6 will result in a poor specificity of only 65%, significantly lower than that of the TCB-_s_. There are many models of transcutaneous bilirubinometers available that use different algorithms. We do not think our findings can be applied to others. The BiliChek® we used was the original model, which may have been continuously upgraded. Here, we recommend validating the TCB readings against TSB for clinical practices that plan to adopt TCB as the tool for NJ management.

When comparing the two skin sites, the study demonstrates that Bilicheck® gives more accurate readings of TCB-_s_ than the TCB-_f_. Based on Figure 6, TCB-s demonstrated better sensitivity and specificity and is considered more optimal for clinical applications. Examining the same neonatal population at both sites allows for comparing the threshold value for admission and checking the TSB levels. Additionally, by treating it as a repeated measure, the analysis gains increased power, resulting in clinically reasonable outcomes.

Ambient light might explain the differences between TCB-_f_ and TCB-_s_ (20). While it is not a common practice to cover the face of neonates, a layette always covers the chest. We hypothesize that the forehead's exposure to ambient light for extended periods may result in an underestimation of TCB-_f_ compared to the TCB-_s_, which is less affected. The influence of ambient light is probably why the manufacturer of BiliChek® markets the BiliEclipse, which can be applied to the forehead to shield the ambient light.

The factors that interfere with the accuracy of TCB measurements are believed to be race, GA, and BW (13). The Bilicheck® has been promoted with the unique advantage of isolating the light absorption of bilirubin from other factors such as hemoglobin, melanin, and dermal thickness (13). In the present study, when adjusting for various factors, such as hours after life, GA, sex, and race, both forehead and chest, TCB readings remained significantly positively correlated with TSB. These findings confirm the device's suitability for varying populations, supporting the widespread clinical applicability of the device. In this cohort, no correlation exists between race (skin color) and the TCB at both the forehead and sternum sites.

Considering patient safety and the potential of medicolegal liability events of adverse patient outcomes, TSB still needs to be obtained to ensure no patients are missed. Our data (Figures 5C,D) showed TCB readings are less reliable when TSB levels are higher than 8.4 or 9.7 for TCB-_f_ and TCB-_s_, respectively. Similar findings have previously been reported (21, 22). For individuals identified as at-risk with NJ, recommending an additional TSB test can help to improve clinical decisions. Our data recommended readings above 12.6 and 14.6 for TCB-_f_ and TCB-_s_, respectively, a reasonable threshold for further TSB measurements.

The results could not be published due to the lack of an adequate statistical method for analyzing neonates having multiple visits, hence the delay. There are a few limitations of the study to note. First, the study's data collection occurred in 2005–2006, and the BiliChek® was the oldest model. AAP has published the revised NJ guidelines in 2022 (9). Other than gestational age and race, we did not record other risk factors Secondly, our original QA/QI project did not register the etiology of NJ and the risk factors such as blood type, hemolytic disease, trauma, breastfeeding, and history of siblings’ NJ that affected the clinical decision. Without that information and identifiers, we could not retrospectively determine which neonate(s) should be admitted for NJ management as thresholds are risk factor adjusted (9). We thus missed the opportunity to analyze our data according to the 2022 AAP guidelines. Thirdly, race was assigned based on maternal records, which might not reflect true skin tone.

## Conclusion

TCB is undoubtedly a convenient tool for following the progression of NJ, especially for busy clinical practices. Race and skin tone do not seem to affect the TCB readings in our study. Although TCB-_s_ performed better than TCB-_f_ in our research according to the 2004 AAP guidelines, clinical decisions based solely on either may lead to many mistakes. Due to the retrospective nature of this study, without risk factors being recorded, we missed the opportunity to re-analyze our data according to the 2022 AAP guidelines. We caution clinical practices to have their calibration study before implementing TCB as the tool for NJ management, as our data show that TCB tends to underestimate the severity of NJ. Such QA/QI projects should follow the newest AAP guidelines.

## Data Availability

The raw data supporting the conclusions of this article will be made available by the authors, without undue reservation.
